# Acute Delirium due to Parenteral Tramadol

**DOI:** 10.1155/2013/492685

**Published:** 2013-06-06

**Authors:** Soumitra Ghosh, Supriya Kumar Mondal, Arnab Bhattacharya, Sahoo Saddichha

**Affiliations:** ^1^Department of Psychiatry, Silchar Medical College, Assam, India; ^2^Central Institute of Psychiatry, Ranchi, India; ^3^Department of Psychiatry, University of British Columbia, Vancouver, BC, Canada

## Abstract

Tramadol is a widely used medication by physicians and is held to be a safe analgesic. It has been claimed to be helpful in the elderly and hepatic and renally compromised subjects. We would like to report a case of delirium in a middle-aged female with acute pain abdomen on tramadol while being treated in the surgical unit. The patient developed alteration in the level of consciousness and cognitive deficits following injectable dose of tramadol. She was diagnosed as a case of tramadol-induced delirium. The patient improved spontaneously after stoppage of tramadol injection. The probable mechanism for development of tramadol-induced delirium is discussed along with clinical implications.

## 1. Introduction

Delirium is an acute confusional state, commonly seen in clinical practice but rarely precipitated by drugs. Tramadol hydrochloride, commonly used as an analgesic, can be an offending agent, and severe delirium can be precipitated by a single dose of tramadol. We report a case of tramadol-induced acute delirium which occurred in a surgical ward and was managed after its early recognition and prompt stoppage of the drug as well as appropriate psychiatric intervention.

## 2. Case Report

 A 55-year-old lady was brought to the emergency and admitted with history of acute abdomen. Ultrasonography of abdomen report suggested cholecystitis with cholelithiasis and small renal calculi. Her routine blood counts, renal and liver profiles, and biochemistries were unremarkable, and chest skiagram and electrocardiogram revealed no abnormality. Conservative management was initiated with intravenous fluids and injections of Ceftriaxone 1 gm and tramadol hydrochloride 100 mg twice daily. Within half an hour of receiving tramadol, she developed confusion and violent behavior, irrelevant talk, and inability to recognize family members. These symptoms persisted for the next six hours till she was sedated with injectable Lorazepam 2 mg. The next morning she got another dose of tramadol for pain relief and again had similar confusional behavior. Due to the previous features, a psychiatric consultation was asked for the patient. Mental status examination revealed a delirious state with disorientation and presence of visual hallucinations. She was diagnosed to be suffering from tramadol-induced delirium. Tramadol injection was stopped but Ceftriaxone continued. Emergency blood glucose, liver and kidney profiles, electrolytes, and thyroid profile were found to be within range. The patient continued on antibiotics and intravenous fluids and attained symptomatic relief. After five days she was discharged from hospital, and during this time she was fully lucid and never had another episode of confusional behavior. 

## 3. Discussion

Our patient had features of delirium, namely, fluctuating levels of consciousness, agitation, confused behavior, irrelevant speech, inattention, disturbance in orientation, and perceptual abnormalities. Multiple factors might contribute to this in a surgical patient like metabolic disturbances, rise of temperature, dehydration, or medications. But on examination she had no feature of dehydration or metabolic derangement, and she was a known nondiabetic and nonhypertensive with no past history of thyroid disease nor any past or family history of psychiatric illness. Moreover, her antibiotic was continued after stoppage of tramadol without any further development of symptoms. So it can be speculated that these factors were unlikely to be a contributing factor in the development of her delirious state. Also delirium developed each time in this patient when she was twice injected with tramadol hydrochloride, and there was a definite temporal correlation (delirium occurred about 30–45 minutes after a dose of tramadol) between the injection and the behavioral abnormality on rechallenge. A Naranjo nomogram score of 9 was obtained which suggests a highly probable cause that it was induced by tramadol [1]. 

Tramadol hydrochloride is a synthetic codeine analog that is a weak opioid receptor agonist and in the treatment of mild-to-moderate pain is as effective as morphine or meperidine. The most common adverse effects are nonspecific central nervous system effects and signs of incoordination [2]. Two reports of tramadol-induced delirium following long-term use of the drug have been observed earlier. However, those cases occurred in the geriatric age group and had a chronic pattern of recurring confusional behavior over a period of two years [3]. Recently one case of delirium following single dose of tramadol was reported in a patient with neurocysticercosis (NCC); however, the involved subject was also concomitantly on ranitidine, steroids, and phenytoin, all of which may give rise to behavioral abnormalities [4]. Moreover, since the subject in that report was already having NCC, he was prone to develop behavioral abnormalities by virtue of his cerebral pathology. But our subject did not have any known significant prior medical comorbidity by which she could have been vulnerable for delirium. 

 The exact mechanism of how tramadol causes delirium is not known, but the probable causative factor is thought to be high plasma level of the metabolite O-desmethyltramadol [5]. O-Desmethyltramadol has an inhibitory effect on M1 and M3 muscarinic receptors and inhibits cholinergic transmission as demonstrated in Xenopus Oocysts expressing cloned M1 and M3 receptors [6]. Thus, inhibition of the muscarinic receptors may induce an anticholinergic confusional state which could be a putative mechanism to explain the delirium in our subject. Other possible mechanisms include the +ve enantiomer inhibiting serotonin reuptake and the −ve enantiomer binding to *α*2 adrenergic receptor and inhibiting norepinephrine (NE) reuptake [7]. Thus, the racemic mixture of tramadol may lead to excess of serotonin and NE, perturbations of which could have been additional pathways to the genesis of delirium in the patient.

In conclusion, tramadol, which is a commonly used analgesic in surgical settings, has the potential to lead to delirium. It is important for surgeons to have a high index of suspicion in order to be able to recognize promptly this uncommon but serious adverse effect of the drug and withdraw the offending agent along with having effective liaisoning with their psychiatric colleagues for appropriate management. 
